# Intersectoral Planning for Public Health: Dilemmas and Challenges

**DOI:** 10.15171/ijhpm.2018.59

**Published:** 2018-07-01

**Authors:** Ellen Strøm Synnevåg, Roar Amdam, Elisabeth Fosse

**Affiliations:** ^1^Faculty of Social Sciences and History, Volda University College, Volda, Norway.; ^2^Department of Health Promotion and Development, University of Bergen, Bergen, Norway

**Keywords:** HiAP, Healthy Public Policy, Governance, Collaborative Planning, Municipality, Norway

## Abstract

**Background:** Intersectoral action is often presented as essential in the promotion of population health and health equity. In Norway, national public health policies are based on the Health in All Policies (HiAP) approach that promotes whole-of-government responsibility. As part of the promotion of this intersectoral responsibility, planning is presented as a tool that every Norwegian municipality should use to integrate public health policies into their planning and management systems. Although research on implementing the HiAP approach is increasing, few studies apply a planning perspective. To address this gap in the literature, our study investigates how three Norwegian municipalities experience the use of planning as a tool when implementing the HiAP approach.

**Methods:** To investigate planning practices in three Norwegian municipalities, we used a qualitative multiple case study design based on face-to-face interviews. When analysing and discussing the results, we used the dichotomy of instrumental and communicative planning approaches, in addition to a collaborative planning approach, as the theoretical framework.

**Results:** The municipalities encounter several dilemmas when using planning as a tool for implementing the HiAP approach. Balancing the use of qualitative and quantitative knowledge and balancing the use of structural and processual procedures are two such dilemmas. Other dilemmas include balancing the use of power and balancing action and understanding in different municipal contexts. They are also faced with the dilemma of whether to place public health issues at the forefront or to present these issues in more general terms.

**Conclusion:** We argue that the dilemmas experienced by the municipalities might be explained by the difficult task of combining instrumental and communicative planning approaches because the balance between them is seldom fixed.

## Background

Intersectoral action is often presented as essential to improving population health and health equity.^1^ This belief is based on perspectives of health being influenced by social determinants found mainly outside of the health sector^2^ and the presentation of public health as a wicked problem.^3^ In recent years, different intersectoral approaches have been used to overcome the constraints of organizational and governmental silos, resulting in the promotion of coordinated actions for health.^4,5^ One such approach is the Health in All Policies (HiAP) approach developed by the World Health Organization (WHO). HiAP is an approach to public policies that systematically takes into account the health implications of decisions made across sectors at all levels of policy-making in order to improve population health and health equity.^5^ Norway has embraced the HiAP approach and made it one of five main principles underpinning the Norwegian Public Health Act (NPHA)^ [1]^ introduced in 2012. By introducing the act, the government asserts population health and health equity as a whole-of-government responsibility,^6^ thereby making administrative and political leadership accountable. As a forerunner in the implementation of the HiAP approach,^7,8^ the Norwegian government (and the NPHA) introduce planning as a tool for securing intersectoral responsibility and political anchoring.^9^ The act obliges municipalities to integrate public health concerns into their planning and management systems when developing local public health policies. For example, municipalities should produce health overviews containing information regarding local health status and local determinants for health, which in turn should form the basis for further planning and action.

Norwegian municipalities are generally responsible for public health and for using planning as tool for implementing a HiAP approach. However, the 422 Norwegian municipalities vary widely in terms of their size, geographical conditions, resources and political and administrative organization. Their prerequisites for meeting local needs also vary. Within a framework of multilevel steering mechanisms, Norwegian municipalities also have a dual role. On the one hand, the municipalities are agents for the welfare state in following the NPHA and implementing national policy goals. On the other hand, they form local independent democratic arenas to meet local preferences and needs.^10^ Implementation of the NPHA mainly involves using soft forms of regulation (such as guidance and education programmes), thereby giving municipalities room to decide how their planning should be conducted.^11^

This self-regulating management means that Norwegian municipalities must manoeuvre the diverse academic landscape of different planning perspectives. In this paper, we bring the debate over what planning is and how it should be done into the discussion and explanation of the municipalities’ experiences when using planning as a tool for implementing public health policies. Traditionally, there have been heated debates over the knowledge foundation of planning and how to proceed from knowledge to action. In many ways, these debates have polarized into two separate and disparate discourses. One side considers planning an instrumental, technical and rational activity where action is separate from knowledge and planners are viewed as experts using top-down approaches. The other side, however, sees planning as a communicative action whereby a variety of different forms of knowledge and action are tied together and planners are viewed as facilitators who use bottom-up approaches to promote reflection and construction.^12^

However, several contemporary scholars^13-18^ have also weighed in on these issues. Davoudi pointed out that despite the extensive critique of planning based on an instrumental rationality, this discussion “keeps creeping back into policy rhetoric, albeit dressed up in new vocabularies such as evidenced-based planning.”^14^ With the upsurge in evidence-based planning, she argued that although it has been widely discussed, specifying the knowledge–action relationship in planning and describing its nuances and challenges remains an important endeavour. According to Innes and Booher,^18^ planning theory has become a set of dividing discourses where people talk past each other and the discussions represent dichotomies that seem incompatible. However, similar to our view in this area of discussion, some scholars (in line with Habermas^19^) have attempted to lessen the discrepancies between the instrumental/rational and communicative/relativistic approaches to planning, promoting them as compatible and complementary and finding pragmatic ways of combining them, thereby representing a collaborative planning tradition.^13,18,20^

In this paper, we define planning as the act of linking knowledge to action in the public domain.^20^ Here, planning is acknowledged both as bureaucratic action and as a political process. This broad conception of planning therefore refers to the activities that facilitate the development of public health policies in general. This means that many professionals at different levels and in different departments in the municipal organization are planners—not just the individuals who write planning documents per se. Nor is planning for public health limited to the physical planning tradition that focuses on land use and its consequences for health—it also involves social and societal elements. In line with Friedman’s^20^ definition of planning, planners engage in one or several of the following activities: defining problems, modelling and analysing situations, designing potential solutions in the form of policies and plans, etc and carrying out evaluations. With regard to the implementation of the NPHA, planning means the processes of analysing situations by producing health overviews, designing potential solutions by making goals and objectives meeting their challenges and needs, implementing actions to fulfil their goals and evaluating their actions.^6^

Earlier research suggests that the implementation and development of the HiAP approach is challenging.^21-23^ Furthermore, scholars have advocated for more research on the intersectoral policy process itself in meeting these challenges.^4,24,25^ In this paper, based on the view of planning as a policy process,^13^ we use a planning perspective as an approach to investigate the implementation of HiAP as a policy process and the policy integration between public health and planning. Several prior research studies used a policy approach at the local level to investigate this integration. However, previous international studies largely consisted of evaluation studies from the Healthy Cities Network, focusing mostly on spatial planning.^26-30^ Previous Norwegian/Scandinavian integration studies used the planning approach more generally, which included societal planning as well. These studies also used different theoretical perspectives, for example, institutional approaches,^7,31^ governance perspectives,^10^ multiple policy streams models^32^ and more empirical approaches.^33^ However, research studies combining communicative and instrumental planning perspectives, discussing what planning is and how it should be done, have been difficult to find.

The aim of this paper is to address the aforementioned knowledge gap by using a planning theory perspective to analyse the integration of planning and public health and to understand the intersectoral process itself when the HiAP approach is implemented at the municipal level. To achieve this aim, we conduct case studies in three Norwegian municipalities as a way to investigate the use of planning as a tool for implementing the HiAP approach by discussing the following question:

*How do the municipalities experience the use of planning as a tool when implementing the HiAP approach?*

The HiAP approach includes both a whole-of-society approach and a whole-of-government approach. However, this paper focuses on the whole-of-government approach only, by investigating the efforts to implement a multisectoral approach for health at the government level, representing actions across government sectors such as health, education and transport, etc.^34^ The whole-of-society approach, representing actions across sectors such as intergovernmental organizations, academia, the private sector, the voluntary sector and civil society, etc, is therefore excluded from this paper.

## Methods

### Design

A qualitative multiple case study design was chosen to obtain an in-depth understanding of planning as a social process.^35^ Three Norwegian municipalities were strategically selected based mainly on their relatively extensive experience integrating public health policies into local planning and management systems. This information was gained through different means: (1) a national survey on the implementation of public health policies in all Norwegian municipalities^36^; (2) a national supervision conducted by the county governor; (3) a webpage containing municipalities’ experiences with the implementation of national public health policies; and (4) information from regional public health advisors. Due to the demographic variations between Norwegian municipalities, the three municipalities selected were chosen as to represent different geographical areas and different population sizes. By investigating the use of planning to implement HiAP as common phenomenon across different types of municipalities, we aim to emphasize results that appear despite their differences, thereby promoting transferability.

Municipality 1 represents a small city on the west coast of Norway with a population in the range of 3500–12 000 inhabitants, with medium to low offers of services and public institutions and categorized as a municipality with a moderately sized amount of disposable income. Municipality 2 represents a small city on the west coast of Norway with a population in the range of 5000–25 000 inhabitants, with medium offers of services and public institutions and categorized as a municipality with a moderately sized amount of disposable income. Municipality 3 represents a medium-sized city on the east coast of Norway with a population in the range of 35 000–150 000 inhabitants, with medium to high offers of services and public institutions and categorized as a municipality with a low amount of disposable income^ [2] ^.

### Data Collection

This multiple case study is based on 30 interviews with municipal employees and politicians from the three selected municipalities. The informants were selected due to their positions and experience with implementing public health policies at a strategic level in their municipal organizations and based on the advice of public health coordinators. Due to different forms of administrative organization within the municipalities, the informants selected varied. The 30 interviews, conducted with 31 informants (13 women and 18 men), included a total of 3 chief executive officers, 3 mayors, 3 politicians, 3 public health coordinators, 2 planners, 2 district medical officers/municipal doctors, 1 public health nurse, 1 advisor, 3 leaders each from the departments of health, education/childcare, technical affairs and culture and 1 leader from the department of development. This relatively large sample was selected to include informants with experience working in different strategic areas at the municipal level in order to gain insight from both informants working systematically to implement the HiAP approach in the municipality as a whole and from leaders representing the different departments, respectively.

Twenty-eight face-to-face interviews and 2 telephone interviews were conducted and recorded during the May 2015 to October 2015 period, mostly at the informants’ offices, lasting from 45 minutes to 2 hours. The interviews were then transcribed by the primary author and by a professional transcribing company. The interviews were based on a semi-structured interview guide containing three main themes: public health terminology, internal processes of intersectoral action and planning and legitimacy of public health policies. This paper mainly presents answers to questions about intersectoral action and planning, where the informants were asked to share their experiences with their efforts to promote intersectoral action across municipal departments and how they use different plans and planning processes to implement their public health policies. Plans and processes of interest included health overviews, public health plans, municipal master plans, action plans, reporting systems and health impact assessments (HIAs), for example.

To prepare the interview questions and to supplement interview data the main author read through the three municipalities’ master plans, action plans/economy plans, annual reports, health overviews and public health plans (if applicable). This reading of documents did not involve separate analysis with separate results.

###  Analysis

Interviews were analysed inspired by Braun and Clarke’s^37^ thematic analysis. First, the material was transcribed, read through and initial ideas were noted. Second, the dataset was coded in a systematic fashion, where codes represented interesting features or meaningful units of data that were relevant to the question of analysis. Then, codes were grouped into potential themes, and themes and codes were reviewed and redefined in relation to the theoretical framework. Themes were organized in thematic maps with superior and subordinate themes/codes and renamed. Finally, detailed analyses of the themes were written up and suitable quotes were selected. When the results section was written up, the themes and codes were reviewed and the text revised to ensure conformity between the materials and the text. The analysis was performed using a hybrid/abductive approach with an inductive starting point, representing a zigzag movement between the theoretical framework and the empirical material.^38^ NVivo 10 software was used to organize the analysis.

Other methods of analysis were also tested, like Attride-Stirling’s^39^ thematic network analysis. However, the organization of the codes into three specific layers (basic themes, organizing themes and global themes) did not align with our particular codes and themes. Instead, Braun and Clarke’s steps of analysis were considered more suitable. A comparative approach was also considered; however, because few obvious differences were found, a more general cross-sectional approach was favoured.

When analysing and presenting the results in this paper, we used the aforementioned dichotomy and debate over instrumental and communicative planning to help describe the informants’ experiences when using planning as a tool to implement a HiAP perspective. Furthermore, when discussing their experiences in more detail, we used Davoudi’s^14^ collaborative planning approach and arguments about planning as the practice of knowing. Specifically, we used her claims about the four characteristics or properties of planning. First, she claimed that planning is *distributed and collective*. This means that judging how to plan for public health is a social process where understanding and ownership emerge from practical collaboration and reflection and where all judgments are tested against the opinions and reflections of other individuals who cast judgment. She argued that the discussions are informative, educational and identity-building^40^ and create common or collective understanding. Second, she described planning as *situated and provisional*, representing an ongoing negotiation between the actors and their settings. Planning is situated in time and space and is specific to a particular context; however, it is also provisional, meaning that the context itself is constantly developing. Third, she claimed planning to be *contested and mediated*, arguing that knowledge and power are mutually dependent. Views of reality reflected in planning for public health are not only cognitive constructs but also instruments of political power.^41^ By defining the truth of how something should be done, what matters or how things should be understood, one exercises power. Power is exercised in different ways, for example, through systems of rules, regulations and procedures, or through forms of representation, such as language, signs, metaphors or symbols. Finally, Davoudi^14^ argued that planning is *pragmatic and purposive.* Planning is pragmatic in that it is more concerned with the consequences of action than with the actor’s intentions^40^ and it is purposive in that it involves the practical judgment of balancing what is intended and what works. She also stated that planners act differently and have different understandings of what planning is or what should be done. Although diverse perspectives and opinions can be sources of conflict, they can also promote innovation and transformation. The characterization of planning as pragmatic is then essential for handling the diverse aspects of planning.

The discussion in this paper builds on Davoudi’s^14^ four properties of planning. Planning is presented as the practice of knowing and as a dynamic process with the following characteristics:

- Distributed and collective
- Situated and provisional
- Contested and mediated
- Pragmatic and purposive

## Results

When using planning as a tool for implementing the HiAP approach in their municipal organizations, the informants experienced several dilemmas that reveal the complexity and diversity of the public health field. The dilemmas represent foundational questions in the planning tradition: what type of knowledge should form the basis for planning public health actions? Furthermore, how should one proceed from knowledge to action? Discussions on these questions seem to be related to the different rationales guiding planning and public health practice, reflecting arguments based on a communicative rationality on the one hand and an instrumental rationality on the other.

### Dilemmas: The Knowledge Basis of Planning Practice

One dilemma identified by the informants in this study was balancing the use of qualitative and quantitative knowledge as a basis for action and practice. The informants pointed out the need for quantitative knowledge representing numbers, trends and explanations of cause and effect when describing public health and its determinants. Some informants favoured this type of knowledge and expressed scepticism towards more qualitative forms of knowledge, representing the local actor’s own experiences of public health and its determinants. However, several informants emphasized the importance of supplementing the quantitative (more instrumental) knowledge foundation with qualitative knowledge. They argued that qualitative knowledge adds nuance to the information gathered about health and its determinants and that the two forms of knowledge need to be combined. For example, when describing investigating the living conditions in one municipality, the leader of the cultural department expressed that the quantitative and qualitative information differed and sometimes even represented contrary results:

*“I’m sceptical of the investigations of living conditions that we use as a basis for prioritization in our municipality. They are based on predefined criteria and only measure hard data. We find that the inhabitants’ experienced life expectancy often represents completely different and even contrary results. We need something that complements and supplements these investigations”* (Leader of the cultural department, Municipality 3).

With regard to the implementation of the HiAP approach, the informants highlighted the importance of a diverse knowledge foundation that is based on both instrumental and communicative forms of knowledge for later success in transferring this knowledge into action.

#### Dilemmas: Transferring Knowledge Into Action

##### Balancing Structural Procedures and Dialogue-Based Processes

Other dilemmas identified by the informants in this study are part of the debate about whether to use more instrumental or communicative procedures in the transfer of knowledge into action when planning for public health. For example, the municipalities use different types of procedures, methods and actions, balancing the use of dialogue-based processes where they arrange for people to meet in dialogue and reflection and more controlling, instrumental structures. On the one hand, using more instrumental structures and instruments is promoted in all three municipalities. They use structural elements, routines and documents in municipal planning and management systems at different levels. For example, public health goals, visions and strategies are included in planning documents at different levels and integrated into budgets/economic plans. Furthermore, HIAs are a routine part of administrative procedures and public health actions are included in annual reports. There are several intentions behind the use of these structural instruments: promoting coherence and intersectoral ownership, securing knowledge-based public health practice, reaching politicians and increasing awareness, authority and status. For example, one leader in the health sector emphasized how integrating public health concerns into planning documents gives them focus, authority and status:

*“The entry ticket for generating focus on something is to get it into formal municipal documents. The municipal master plan, action plans, budgets and annual reports are important tools for getting attention. What’s included here automatically gets status, and that’s what we have done”* (Leader of the health department, Municipality 2).

The informants explained that the structural instruments are essential. According to some informants, including public health intentions and goals in plans and administrative procedures is not very hard to do in principle. However, they questioned the use of structural instruments because this does not necessarily lead to action. Even though municipal plans, routines and systems are established for transferring knowledge into action, these structures do not necessarily govern municipal practices. The informants stated that in reality, budgets, management by objectives and KOSTRA numbers (a municipality-/state-reporting system that provides quantitative information on municipal activities), rather than goals and visions, for example, are what determine municipal priorities in the municipal master plans.

Informants are intimidated by the emphasis on economics and efficiency in policy development. One informant explained the situation as follows:

*“For example, public health issues are clearly seen in our municipal master plan. And that’s great! The problem is that the municipality is not governed by the municipal master plan—it’s governed by numbers. And then, what’s written about public health doesn’t really mean much after all”* (Public health coordinator, Municipality 2).

In continuation of this theme, the informants questioned whether there is any point to integrating public health policies into these superior planning documents when they are not genuine management mechanisms. Other informants further problematized this issue by arguing that these instrumental procedures for management (eg, KOSTRA, management by objectives and budgets) are incompatible with some of the characteristics of public health. For example, one public health coordinator highlighted the need to integrate public health concerns into the municipal economic plan but expressed that this was difficult to do because of public health’s long time frame and qualitative indicators based on values and ideology, not numbers or logic. This informant stated the following:

*“This management by objectives used in the municipalities just does not fit… Firstly, this is long-term work. Secondly, there are indicators that cannot be measured in numbers… Either we have to change the system of management—and that might be a solution—or else we have to adapt the management by objectives system, customized to the use of qualitative indicators”* (Public health coordinator, Municipality 2).

The use of structural instruments when implementing the HiAP approach was widely employed in all three municipalities. However, several informants noted the need for less instrumental and more process-oriented planning procedures. Using planning methods based on dialogue, reflection and participation means that value-based knowledge can be included. In addition, the informants highlighted the importance of using dialogue and participation to promote understanding and ownership of public health policies. For example, one municipal doctor attributed the success of implementing the HiAP perspective to using open dialogue and promoting the broad participation of professionals from different departments within the organization:

*“We have experienced building this together, that we have ownership… This is related to the questions: Who is engaged? What are the opportunities for dialogue? Should there be a narrow or a wide dialogue? I believe that there are no other questions that should be as open and broad as the questions about public health issues”* (District medical officer/Municipal doctor, Municipality 1).

The promotion of participation was detected in varying degrees in public health planning processes in all three municipalities. The informants promoted participation as a precursor to ownership and identified ownership as being essential for a plan and a product to lead to action. Some informants promoted the planning process based on broad participation as a goal in itself, as opposed to the planning document alone. One public health coordinator described the situation as follows:

*“The process of making our public health plan was supposed to take some time. This was not a fast-paced project. This was the first public health plan and we needed to build knowledge about public health in our organization. We wanted the entire municipal organization to be included in producing the plan—for all to be heard and to create ownership. And then you get a better product”* (Public health coordinator, Municipality 3).

When promoting these dialogue-based processes, the informants acknowledge the need for soft skills, setting aside their roles as experts. However, the informants acknowledge this as a challenging task. Being facilitators instead of experts and sources of truth might represent a new and different role for some municipal professionals. One public health coordinator described the situation as follows:

*“With my background, I believe that the communicative planning approaches are quite challenging for us. These concerns [are as follows]: What roles do we have? When are we authorities? I tend to think that I know best, and I believe that many in these types of positions do. So this feels a bit uncomfortable, of course; however, we are told that this works”* (Public health coordinator, Municipality 3).

The informants’ experiences and dilemmas of whether and when to use structural and dialogue-based methods and procedures seem to reflect the ongoing debate about communicative and instrumental planning.^12^ On the one hand, they advocated using instrumental structures and procedures for promoting, for example, awareness, authority and status. However, they also acknowledged the shortcomings of these methods. On the other hand, the development of more communicative planning procedures that facilitate dialogue and participation, thereby promoting meaning and reflection, was stated as essential for understanding and ownership. However, they also acknowledged the challenges this perspective involves. When implementing the HiAP approach, all three municipalities used both perspectives. One leader expressed a need to balance the different planning approaches. The leader suggested a combination of integrating public health policies into documents and administrative procedures with more interactive methods based on dialogue, process and participation.

##### Balancing Power and Power Relations

Some of the contradictions between instrumental and communicative planning practices are also reflected in the issue of power. For example, a few informants experienced the implementation of the HiAP perspective to be somewhat imposed or enforced, especially with the use of instrumental methods such as HIAs. One leader stated the following:

*“In some cases, some of us might feel that it gets a bit imposed and is unnatural to demonstrate public health issues all the time—that it is some sort of form that we’re incorporated into. We’re supposed to think about the consequences for public health, but that does not always feel natural”* (Leader of the cultural department, Municipality 1).

The informants pointed out the risk of some sort of health dominance or coercion occurring when the HiAP perspective is adopted in municipal organizations. The issue of the balance of power represented a dilemma for the informants. On the one hand, the informants acknowledged the possibilities of promoting awareness when instructing and pointing actors in a certain direction. On the other hand, they acknowledged the potential for conflict when telling others what to do. Informants emphasized the need for soft skills as humility and respecting others’ knowledge and competence. For example, coordinating teams and public health coordinators should be facilitators who are available as discussion partners; their job is to promote finding a sort of common ground, not to define best practice. One leader described the situation as follows:

*“When we arrive, we represent the executive officer, the superior system. It’s important to develop a role that makes people respond, “So nice that you’re coming. Please help us!” I believe that the worst [thing] you can do in an established organization structure is to create an umbrella [new organization] above. This is raising a red flag for Norwegians. You just don’t do this”* (Leader of department for development, Municipality 3).

The informants highlighted the importance of acknowledging the issue of a power imbalance and its possible consequences when implementing a HiAP perspective. When instrumental procedures and demands are used instead of dialogue, understanding and respect, there is a risk of forcing every municipal actor into becoming a public health worker (ie, failing to respect other disciplines’ professional knowledge and identities).

##### Balancing Activities in Different Municipal Contexts

Another dilemma experienced by the informants was balancing the public health action on the different hierarchical levels in the municipal organizations, which represent different contexts. The informants acknowledged the superior system level of the organization as important in coordinating a holistic approach for their municipal public health policies. However, they also acknowledged the role of the operative level for the implementation of public health actions. For example, the informants discussed their public health goals and the need to balance goals on different levels. The use of superior and overall goals and visions at the system level was highlighted as necessary for establishing the wider context (ie, for framing the municipal activity). However, these goals and visions need to be communicated and converted into clear and concrete goals, closer to action at the operative level.

One leader argued linking the action at these two levels together. This informant pointed out the need to organize meeting points or arenas, which he called “cross-sectoral space for discussion,” where representatives from the superior system level in the municipality can meet and discuss with the representatives from the operative level. Within this space, he highlighted the need to work in “sine waves” between the different levels, representing an undulating movement up and down between the executive and operative levels. By doing this, the representatives from the different levels might gain a better understanding of the arguments and ways of working and thinking of the other levels, and thereby acquire holistic and contextual insight and understanding. Although this leader stressed the importance of individuals from the different levels meeting, the informant also acknowledged several challenges. For example, he recognized the difficulties some might have when working from more communicative perspectives and he worried that his municipal organization lacked professionals with the dialogue and processual competence needed in the cross-sectoral space for discussion:

*“We [at the executive level] need to enter the operative world, and that’s a rather different world. It’s very comfortable dealing with theory, very comfortable! Therefore, you challenge the academic comfort zone. Some handle this just fine; however, others are more reluctant. In general, I believe that the municipality needs a combination of employees with theoretical knowledge and employees who manage being in this [cross-sectoral] zone. And yet this [need] is unfulfilled”* (Leader of department for development, Municipality 3).

Several informants stressed the importance of seeing their work in a broader and superior context. However, the reflections of this leader also emphasized the subsequent challenges associated with the more communicative procedures needed to gain contextual understanding.

##### Public Health: Specifically or Generally?

Another dilemma faced by the municipalities is whether to make public health structures and processes distinct from other municipal structures and processes or to integrate them into those that already exist. This dilemma manifests itself differently in different municipalities. For example, one of the municipalities has a distinct public health plan, whereas the other two municipalities integrate public health goals and visions into their master plans. Furthermore, two of the municipalities use separate public health working groups; however, one uses an existing “planning group” for raising public health issues. In addition, the process of gaining knowledge of health conditions and determinants, in accordance with the NPHA, is carried out as separate “public health processes” in all three municipalities, with the intention of integrating them into more general planning processes eventually.

The informants noted there are challenges and benefits associated with both alternatives. Some informants experienced the action of creating distinct public health processes as problematic because this makes public health a specific field of interest, which is not in accordance with the HiAP approach. One mayor stated the following:

*“I believe that it is important to anchor this at the highest level in the municipal master plan, not making public health something special… The day you make a specific plan, you isolate this to a specific field of interest”* (Mayor, Municipality 1).

However, the informants also expressed the view that distinct processes is beneficial and important for placing public health on the policy agenda, thereby promoting awareness and knowledge about public health. One informant related this to why they chose to make a distinct public health plan instead of integrating public health issues into the municipal master plan:

*“The municipal master plan has a different process; it’s not customized to promote public health issues in particular. We wanted a good and long process where we could discuss public health issues and secure a public health policy that is developed and agreed on by all politicians… We needed to concretize and to promote knowledge”* (Public health coordinator, Municipality 3).

Some informants also suggested that this discussion of whether to make public health something special is important when communicating with public health actors. Some informants experienced a rejection of the responsibility of public health by others, arguing that this cannot be taken on in addition to everything else. Based on this, some informants expressed that public health work should not be communicated as something new or special that is performed in addition to daily tasks. Rather, the mission is to take a more pragmatic approach to make municipal actors do more of what they already do by communicating public health as general tasks that are integrated into what already constitutes their existing responsibilities.

## Discussion

Overall, the three municipalities’ experiences reflect the complexity and diversity of planning practices when implementing the HiAP perspective. On the one hand, the municipalities promote quantitative expert knowledge together with structures, routines and demands that more instrumentally define the truth of how to proceed and understand planning for public health and promoting awareness, authority and status. On the other hand, they promote qualitative experience-based knowledge where knowledge is produced through collective development processes and, setting aside their role as experts, requires soft skills such as humility and respect. Several scholars have argued that it is important to combine different types of knowledge and different procedures and approaches in planning, particularly when planning for complex or wicked problems like public health issues.^13,14,18^ However, combining these approaches might create challenges since the balance between the different approaches is not fixed.^14^ Davoudi’s^14^ characteristics of planning (ie, *distributed and collective*, *situated and provisional*, *contested and mediated*, and *pragmatic and purposive*) will be used to understand some of the dilemmas identified by the municipalities in this study.

### Planning as Distributed and Collective

According to Davoudi,^14^ planning for public health is distributed and collective, representing a social process where understanding and ownership emerge from practical collaboration and reflection. Some informants in this study stressed this perspective by presenting dialogue-based processes as criteria for securing common understanding and ownership of the local public health policies. Dialogue-based processes and procedures for securing multisectoral actions for health have also been described in earlier public health research.^29,42^ For example, in one study, the processes of making city development plans were experienced as being as important as the planning documents themselves.^29^ However, challenges arise when the participative and communicative perspective meets the more instrumentally defined demands of efficiency and economic arguments in municipal organizations. Even though municipal actors promote the collective and participatory aspects of planning, some of the informants in our study expressed that the organizational routines and structures within the municipalities are not equipped to handle this diversity of knowledge. The informants seemed to experience what Héritier and Lehmkuhl^43^ called “sectoral governance in the shadow of hierarchy”, reflecting the challenging act of promoting collective processes when embedded in more hierarchical, fragmented, autocratic or instrumental forms of government. We observed the debate about this issue in several traditions, for example, in collaborative planning,^13,44^ in regard to governance processes in general^42,45^ and in the implementation of the HiAP perspective specifically.^31,46^ According to Hofstad,^31^ the meeting of different rationalities in planning for public health issues might explain some of the difficulties encountered when integrating planning and the health promotion tradition. She claimed that planning’s starting point, which is often economic, complicates its integration with the health promotion tradition, where quick and ready-made answers are impossible.

### Planning as Situated and Provisional

Davoudi^14^ argued that planning is situated in time and space; that is, planning is specific to a particular context. The informants in this study agreed with this argument, stressing the importance of contextual understanding when using planning as a tool in public health work. Carey and Crammond^46^ reached a similar conclusion when their informants experienced the need to break down the social determinants of health, communicating this information in suitable ways within the different government departments. However, planning as a situated action might also explain some of the challenges expressed by the informants in the present study. For example, one informant expressed that municipal employees from the executive system level of the organization might have difficulties understanding public health actions and arguments at the operative level (ie, they view public health work from a more theoretical and superordinate angle). Although challenging (or perhaps for that very reason), the meeting of different types of thought and action based on different kinds of contextual understanding was promoted as important by the informants in this study. Doing so facilitates what one informant called “cross-sectoral spaces for discussion,” for example. Earlier scholars have made similar arguments about interdisciplinarity. According to Buanes and Jentoft,^47^ establishing arenas and meeting points, which promotes being in contact with other departments and gaining knowledge about other departments’ work and realities, is an important element of interdisciplinary success because one gains understanding and knowledge about the overall context of the organization.

Encouraging actors to meet to engage in dialogue and reflection might also have further consequences. According to Davoudi,^14^ planning as practice of knowing is not only situated or contextual but also provisional. This means that public health planning is constructed and constantly changing within a context that in itself is constantly developing. The knowledge and understanding of public health work planners depends on their context. However, when planners adapt or change their understanding, this can prompt them to adapt and change their social or institutional structures further. In this way, actors meeting in the “cross-sectoral space” might promote common contextual understanding and create opportunities for institutional or sociopolitical adaptations or changes to take place in practices, structures, routines and so on.^14^ One informant pointed out the need for institutional change (ie, a measurement system within the municipalities that is adjusted to allow for more communicative approaches and procedures) or the need for a new system altogether.

### Planning as Contested and Mediated

Davoudi^14^ suggested that planning is contested and mediated, arguing that knowledge and power are mutually dependent. Aware of the risks of using power and the need for mediation, several informants in this study promoted planning as contested. However, their experiences also suggested that the issue of power is complicated and varied. According to Davoudi,^14^ power is exercised through systems of rules, regulations and procedures, for example. Some informants in this study viewed the use of systems, rules or structures (eg, HIAs or mandatory annual reporting on health issues) as being imposed or enforced, which in turn hindered creativity. They highlighted the importance of not telling others what to do, not steering their actions and opinions excessively and not defining the truth. Instead of telling others what to do, they suggested that those in the planner role should use soft skills when implementing HiAP. They should respect the knowledge and competence of others, be available as discussion partners, be open to others’ opinions and use this humble approach to create common ground. The use of soft skills when implementing HiAP is only vaguely discussed in the literature; however, soft skills such as humility, trust, openness and patience are recognized as essential by other practitioners.^48-50^ Despite recognizing that structures such as HIAs and annual reporting might be experienced by some as imposed or enforced, the informants in our study also acknowledge that these structures are important implementation and development tools to raise awareness, authority and status. According to Davoudi,^14^ and contrary to the conventional portrayal of rules and regulations as fixed and rigid inhibitors of creativity, they are also dynamic features that might simultaneously represent opportunities, innovation and change.

In planning practice, power is also exercised through forms of representation, such as language, signs, metaphors or symbols.^14^ By using language to name activities and concepts, one somewhat promotes knowledge, defines the truth and exercises power. The municipalities experienced a dilemma over whether to conduct planning processes and structures in the municipalities as specific public health processes (ie, place public health at the centre of attention and refer to those processes and structures collectively as “public health”) or to integrate them into general municipal activities. The informants stated that creating special public health groups, public health plans and public health processes, for example, is beneficial and important when it comes to placing public health on the policy agenda and to promote awareness and knowledge about public health, which is considered essential in the implementation of the relatively new NPHA.

However, the health dominance of the development of the HiAP perspective in the municipal organizations, where health should be integrated into all municipal activities, can be experienced as a threat. Making public health something special by placing it at the forefront can lead to the exertion of some sort of power by the public health discipline. In this situation, public health work might be viewed as a threat to other disciplines (eg, through its taking over of professional arenas), resulting in public health work being viewed with distrust. Other scholars have also acknowledged this issue in public health practice, recognizing the risk of health imperialism.^1,24,51-54^ For example, Breton^52^ argued that health imperialism, where the health sector tries to impose its priorities on other sectors, is a major impediment when implementing the HiAP perspective to promote health. In line with several informants in this study and with more collaborative planning approaches,^13,14^ he stated that all departments need to invest time and energy into their relationships with each other to promote understanding.

In addition, Carey and Crammond^55^ criticized the HiAP approach in general, whereby health is sanctioned as the most important policy. They compared HiAP to the more general perspectives of joined-up government and stated that HiAP might fail when promoting the integration of health into every other sector instead of promoting integration more broadly between multiple departments simultaneously. In line with their argument, one might question whether every sector in a municipality can be expected to operate under the public health banner or, alternatively, whether one should expect some sort of scepticism or distrust. As we understand Carey and Crammond’s^55^ critique, it discusses the underlying assumptions and ambitions of the HiAP approach. What do we aim for in using the HiAP approach? What type of involvement of the different sectors do we wish to achieve? In the literature on HiAP, the terms cross-sectoral and intersectoral seem to be used interchangeably.^5,56,57^ However, we argue that these terms might indicate different ambitions for how to involve different sectors. In this paper, we use the term intersectoral action in line with how we understand Buanes and Jentoft’s^47^ use of the term interdisciplinary. Here, interdisciplinary action means more than just coordinating actions—it includes the act of challenging others’ institutional foundations and learning from each other, resulting in something new, something different or some sort of development. Could some of the criticism directed at the HiAP approach (and the challenges experienced by our informants) be related to the challenging tasks of moving from cross-sectoral towards more intersectoral actions for health? This debate is beyond the scope of this article, however it warrants further academic discussion.

### Planning as Pragmatic and Purposive

The final characteristic of planning put forward by Davoudi^14^ is planning as pragmatic and purposive. This means that planning involves the practical judgment of balancing what is intended and what works. This characteristic might help to understand the need for mediation promoted by the informants in this study, that is, how to handle the health dominance/power imbalance/health imperialism dilemma discussed earlier. Some informants expressed that when communicating the HiAP perspective (ie, when convincing all sectors to accept their responsibilities), they should not present public health actions as something new or as something that the public health coordinator suggests that they do, but rather as work that is already being carried out in the respective departments. Fulfilling the mediating role is essential for planners or other professionals who play a part in public decision-making.^18^ However, according to Holt et al,^58^ this common mediating practice (one frequently carried out by municipalities) can be counterproductive to implementing the HiAP approach. They argued that when public health actions are communicated as “something you will do anyway,” the intersectoral policy tends to favour intermediary determinants for health (eg, behaviour change) and not the causes of the causes (eg, poverty and lack of education). The interventions hereby fail to address the more fundamental social determinants of health. Supported by Steenbakkers et al,^23^ for example, they concluded that rather than considering non-health sectors that do not have health as their central priority problematic, they should be considered representative of the normal state of affairs. The informants’ experiences seem to support the conclusions of Holt et al.^58^ For example, they argued that labelling processes as public health runs the risk of making public health work seem to be an issue for someone in particular and not everyone in general, as promoted by the HiAP approach.

The discussion presented here demonstrates one of several dilemmas experienced in the three municipalities when implementing the HiAP approach, where planning is represented as contested and mediated and as pragmatic and purposive.^14^ On the one hand, implementing pure public health structures and processes can promote awareness and put public health on the political agenda. On the other hand, placing public health at the centre of attention might be considered imperialistic or be met with distrust, and thus be counterproductive for implementing the HiAP approach. We support Holt and colleagues’^58^ pragmatic argument that careful attention and evaluation are essential when health is expressed as an explicit and particular goal (ie, placed at the forefront of particular processes) and when health supports the policies and goals of other sectors in general. We argue that the use of power when planning for public health issues needs to be balanced in the same way that instrumental and communicative perspectives are recognized as complementary and reciprocal.^13,18,43^

## Limitations

In line with the aim of this study, a qualitative case study design was chosen. One limitation of the study is that the findings are dependent on the context, which thereby raises questions about objectivity and generalizability.^35^ However, the design allows us to gain an in-depth understanding of the issue, which produces knowledge that is useful and transferrable to similar situations. Another possible limitation is the lack of in-depth analytical and comparative work conducted on the cases. Still, the chosen analytical approach allows us to present more general and superordinate descriptive information about the dilemmas and challenges faced by the municipalities. Furthermore, the case study involves a mix of voices among the informants, indicating the possible tension between planning as policy (representing the bureaucratic approach to planning) and planning as politics (representing public health and health equity as a political concern). However, the informants were all selected upon the advice of local public health coordinators who were specifically asked for the names of politicians with knowledge of and insight into the planning policies investigated in this study. The questions were also designed to gain insight into bureaucratic processes in particular. Still, these tensions between the different roles among the different informants must be taken into account when considering the validity of the study.

## Conclusion

Based on the results and discussion presented in this article, the debate over the instrumental and communicative planning perspectives continues—at least in terms of public health planning practice. Norwegian municipalities face several dilemmas when planning for public health, reflecting a complex and multidimensional planning practice. The dilemmas and challenging situations experienced in the municipalities might be explained by the difficulty of the task of combining the instrumental and communicative approaches, which stems from the fact that the balance between them is seldom fixed. On the one hand, the municipalities promote the use of quantitative expert knowledge together with structures, routines and demands that more instrumentally define the truth of how to proceed and understand planning for public health. On the other hand, they promote qualitative experience-based knowledge, which is produced through collective processes that include dialogue and participation and require soft skills for their implementation. Managing this balance is especially challenging for municipalities when operating in organizations that favour instrumentally defined government mechanisms, such as management by objectives and economic efficiency, and struggle to accept and facilitate communicative rationalities.

## Ethical issues

The Norwegian Social Science Data Services (NSD) approved the study and the informants provided their informed consent.

## Competing interests

Authors declare that they have no competing interests.

## Authors’ contributions

All three authors (ESS, RA, EF) contributed to the conception and design of the study. ESS was responsible for acquisition, analysis, and interpretation of data, in addition to drafting the manuscript. RA and EF participated with supervision throughout the process and provided critical revision of the manuscript for important intellectual content.

## Authors’ affiliations

^1^Faculty of Social Sciences and History, Volda University College, Volda, Norway. ^2^Department of Health Promotion and Development, University of Bergen, Bergen, Norway.

## Endnotes


[1] In a Norwegian context, the term *public health* represents a broad approach to public health, encompassing traditional disease prevention perspectives, newer health promotion ideologies, and approaches for reducing social inequalities in health.

[2] The classification of the municipalities is based on the following reports:

Gundersen, F, Jukvam, D. Inndelinger i senterstruktur, sentralitet og BA-regioner. Norwegian Institute for Urban and Regional Research: 2013.

Langørgen, A, Løkken, SA, Aaberge, R. Gruppering av kommuner etter folkemengde og økonomiske rammebetingelser 2013. Statistics Norway: 2015


## Key messages

**Implications for policy makers**

- Intersectoral planning involves the challenging task of balancing qualitative and quantitative knowledge.
- Intersectoral planning involves the challenging task of balancing instrumentally defined structures, routines, instruments and so forth, and dialogue-based processes where people meet in dialogue and reflection.
- Intersectoral planning involves the challenging task of facilitating meeting points for municipal actors across departments and hierarchical levels to promote common understanding.
- Placing public health issues at the forefront might elevate status and increase attention, but it might also increase the risk of some sort of health imperialism.

**Implications for the public**

Public health is affected by several different determinants lying outside of the health sector; therefore, different municipal sectors need to collaborate across their borders. This study identifies current dilemmas in intersectoral collaboration. The results of this study are therefore useful for the public and public health in general ensuring that public health actions and considerations reflect the diversity of determinants of people’s health By promoting intersectoral collaboration throughout the municipal sector, public health can be achieved within the various settings in which people work, learn and live, not only through their use of healthcare services.
